# Discovery of Antimalarial Drugs from Streptomycetes Metabolites Using a Metabolomic Approach

**DOI:** 10.1155/2017/2189814

**Published:** 2017-10-16

**Authors:** Siti Junaidah Ahmad, Mohd Badrin Hanizam Abdul Rahim, Syarul Nataqain Baharum, Mohd Shukri Baba, Noraziah Mohamad Zin

**Affiliations:** ^1^Programme of Biomedical Science, School of Diagnostic and Applied Health Sciences, Faculty of Health Science, Universiti Kebangsaan Malaysia (UKM), 50300 Kuala Lumpur, Malaysia; ^2^Institute of Systems Biology (INBIOSIS), Universiti Kebangsaan Malaysia, 43600 UKM Bangi, Selangor, Malaysia; ^3^Department of Biomedical Science, Kulliyyah of Allied Health Sciences, International Islamic University Malaysia, Jalan Sultan Ahmad Shah, 25200 Kuantan, Pahang, Malaysia

## Abstract

Natural products continue to play an important role as a source of biologically active substances for the development of new drug.* Streptomyces*, Gram-positive bacteria which are widely distributed in nature, are one of the most popular sources of natural antibiotics. Recently, by using a bioassay-guided fractionation, an antimalarial compound, Gancidin-W, has been discovered from these bacteria. However, this classical method in identifying potentially novel bioactive compounds from the natural products requires considerable effort and is a time-consuming process. Metabolomics is an emerging “omics” technology in systems biology study which integrated in process of discovering drug from natural products. Metabolomics approach in finding novel therapeutics agent for malaria offers dereplication step in screening phase to shorten the process. The highly sensitive instruments, such as Liquid Chromatography-Mass Spectrophotometry (LC-MS), Gas Chromatography-Mass Spectrophotometry (GC-MS), and Nuclear Magnetic Resonance (^1^H-NMR) spectroscopy, provide a wide range of information in the identification of potentially bioactive compounds. The current paper reviews concepts of metabolomics and its application in drug discovery of malaria treatment as well as assessing the antimalarial activity from natural products. Metabolomics approach in malaria drug discovery is still new and needs to be initiated, especially for drug research in Malaysia.

## 1. Introduction

Over the centuries, natural products including animals, plants, and mineral have played an important role in drug discovery and development for the treatment of human disease [1, 2]. Streptomycetes are Gram-positive bacteria, which are widely distributed in nature and abundant in soil. This group of bacteria produces secondary metabolites such as antibiotics and their derivatives, which have been synthesized biosynthetically from the primary metabolites, have been applied towards combating pathogens and cancer [2, 3]. Despite being used for years in the development of new therapeutic agents, finding potential novel compounds from the streptomycetes metabolites for the treatment of human disease such as malaria remains a great challenge.

Dereplication is a process used in recognising and eliminating the active substances that have already been studied in the early stage of the screening process [4]. This process is considered as a stage following the preliminary screening in discovering new pharmacologically active compounds in an extract from natural products [5]. Recently, metabolomics approach has been applied in the dereplication process [6, 7]. Nuclear magnetic resonance (NMR) spectroscopy- and mass spectrometry- (MS-) based metabolomics approaches are powerful tools that facilitate quick identification of both the targeted and untargeted metabolites present in a complex mixture of crude extract during the screening phase. Hence, these methods eliminate already well-known natural products from further isolation process [8].

Drug discovery from natural product utilizing metabolomics platform gives a significant tool in system biology. This allows researchers or pharmaceutical scientists to gain some insight about new potential pharmaceutical agents and to manipulate the factors within fermentation system in sustainable manner to select a desired metabolite [9]. The present review covers the metabolomics approach in drug discovery of malaria treatment and discusses the potential of natural antibiotic products from streptomycetes.

## 2. Metabolomic Concept

Metabolomics is a systematic, qualitative, and quantitative study of small bioactive molecules in organism, biological fluid, plants, and food matrices at specific time and under specific conditions [8]. This study is about all metabolites set within organism or tissue [10]. Metabolomics is a branch of “omics” technology that focused on high-throughput identification and quantification of small molecules metabolites in metabolome. Metabolome is a set of all small molecules of metabolites that are found in a cell, organ, or organism. These molecules include chemical entities such as peptides, amino acids, nucleic acids, carbohydrate, organic acids, vitamins, drugs, food additives, phytochemicals, and toxin [11].

Metabolites are divided into two types, primary metabolites and secondary metabolites. Primary metabolites are normally produced during the growth phase of the organism as well as during energy metabolism [12]. These primary metabolites such as carbohydrate, protein, amino acids, and fatty acids are vital for the cell and normally involve cell growth, development, and reproduction [13]. Secondary metabolites, on the other hand, are synthesized from the primary metabolites during certain condition and play a role in defence mechanism from pathogen and abiotic stress [14]. These low-molecular-weight metabolites (<3 kDa) comprise a highly valuable class of compounds that can be used in many applications such as drugs (e.g., antibiotics, antitumor agents), agrochemical agents (e.g., pesticides), biofuel (e.g., oleoresin), and food additives (e.g., essential oils) [15]. It has been reported that the microbial secondary metabolites from fungus and bacteria exhibit medicinal value as an antimicrobial agent, sor example,* Streptomyces kebangsaanensis* that produce phenazine [16]. In general, metabolomics could be used to identify and to optimize the production of secondary metabolites. Information gathered from a metabolomics dataset can efficiently establish cultivation and production processes at a small scale which will be finally scaled up to a fermenter system.

## 3. *Streptomyces*

*Streptomyces *is the largest genus of Actinomycetes.* Streptomyces* is Gram-positive aerobic bacteria which produce a network of branched filaments called substrate mycelia and aerial mycelia. Their cell wall contains alanine, glutamic acid, glycine and LL-2, and 6-diaminopimelic acid (*LL*-DAP) which is an amino acid and its nucleus has more than 70% guanine and cytosine (G+C) content [17].

This Gram-positive bacteria produce secondary metabolites which have the potential to be used as source of natural antibiotic such as daptomycin. Moreover, the secondary metabolites produced by* Streptomyces *also have been shown to act as immunosuppressants (e.g., rapamycin), antifungals (e.g., amphotericin B), anticancers (e.g., doxorubicin), and antiparasitics (e.g., ivermectin) [18].

## 4. Bioactive Compounds from Streptomycetes Metabolites

Bioactive compounds from* Streptomyces *spp. have been used as source of natural antibiotic product. From the late 1940s to the 1960s, which is also called the golden age of antibiotics discovery era, many antibiotics were isolated from various* Streptomyces* species and entered clinical use [13]. One of the unique features of the genome in the* Streptomyces* species is the presence of biosynthetic gene clusters which encode enzymes that are involved in the production of secondary metabolites [19]. Examples of bioactive compounds produced by* Streptomyces* sp. are listed in Table 1.

Trioxacarcins are complex antibiotics that were first isolated in 1981 [29]. In later years, [20] has isolated trioxacarcins A, B, C, and D from marine streptomycetes and has tested the compounds against* Plasmodium falciparum* (*P. falciparum*). Out of these four compounds, trioxacarcins A and D show extremely high antiplasmodial activity (with the IC_50_ value 1.6 ± 0.1 and 2.3 ± 0.2 ng/mL, resp.) which is comparable to the most active compound, artemisinin (IC_50_ value 0.7 ± 0.1 ng/mL). Antiplasmodial activity shown by trioxacarcin B (IC_50_ value is 102 ± 4.9 ng/mL) was about 100 times less than trioxacarcins A and D, while trioxacarcin C was nearly inactive (IC_50_ value is >5000 ng/mL) [20].

Coronamycin is a complex of novel peptide antibiotics isolated by Strobel et al. in 2004 [30]. This compound is isolated from endophytic* Streptomyces* sp. from an epiphytic vine,* Monstera* sp., which can be found growing in the upper Amazon region of Peru [21]. Coronamycin demonstrated antiplasmodial activity against* P. falciparum*, with an IC_50_ of 9.0 ng/mL. While coronamycin does not exhibit anticancer activity against breast cancer cell line (BT20), its cytotoxicity to primary human mammary epithelial cell line (HMEC) is comparable to taxol, an anticancer chemotherapy drug [21].

In 2002, Strobel and coworkers discovered a series of unique wide-spectrum antibiotics called munumbicins (A, B, C, and D). Munumbicin D caught the most attention as it shows an activity against the malarial parasite* P. falciparum*, with an IC_50_ of 4.5 ± 0.07 ng/mL [23]. Another bioactive compound which has demonstrated antimalarial activity against* P. falciparum *was discovered in 2003 and has been identified as kakadumycin A. Kakadumycin A isolated from* Streptomyces* sp. NRRL 30566 yielded an IC_50_ of 7.04 ± 0.12 ng/mL when testing against malarial parasite* P. falciparum* [25]. Strobel and coworkers continue to make a discovery and isolated two novel peptides, munumbicins E-4 and E-5, from an endophytic* Streptomyces* NRRL 30562, isolated from* Kennedia nigricans*, snake vine, in the Northern Territory of Australia. These compounds showed an antimalarial activity against* P. falciparum* with IC_50_ values of 0.50 ± 0.08 and 0.87 ± 0.0.26 mg/mL for E-4 and E-5, respectively [22].

Another bioactive compound that has shown an antimalarial activity is Gancidin-W, which has been isolated from* Streptomyces* sp. SUK 10.* In vivo* testing of Gancidin-W on* Plasmodium berghei* NK 65-infected mice showed an inhibition of 80% of the parasite at the concentration of 6.25 and 3.125 *μ*g/kg body weight on final day of the test. In addition, 50% (n-3) of mice treated with Gancidin-W at concentration of 3.125 *μ*g/kg body weight survived until 11 months after inoculation of infection [26].

Bioassay-guided fractionation of the extract from the fermentation broth of* Streptomyces spectabilis *BCC 4785 led to the isolation of metacycloprodigiosin. This compound showed potent in vitro activity against* P. falciparum *K1, with IC_50_ of 0.0050 ± 0.0010 *μ*g/ml, while bafilomycin A_1_ isolated from* Streptomyces* sp. CS showed antimalarial activity against* P. falciparum* K_1_ with IC_50_ value of 0.041 ± 0.010 *μ*g/mL [24].

However, metabolomics approach has not been used or only partially used in all of the drug discovery works mentioned above. It could be because the scientist tends to search for activity of the particular compounds rather than thinking about the redundancy of the compounds. Along the process in bioassay-guided fractionation, the scientist could be missing some of the potentially active compounds due to inability to build in library at the prepurification stage. The use of metabolomics could help prioritize fractions for further purification which could save time and resources in isolating target compound [8].

### 4.1. Screening and Assessing for Antimalaria Activity of Natural Products

For the purpose of assessing antimalarial activity of the drugs, two methods have been developed:* in vitro* and* in vivo* methods. The details of each method are discussed below.

#### 4.1.1. *In Vitro* Study

*In vitro* analysis is comprised of microscopy-based assay (also known as WHO microtest), tritium-labeled hypoxanthine assay (isotopic assays), fluorimetric assay (using DNA-specific dyes), cytometry-based assay, and enzyme-linked immunosorbent assay (ELISA) [31, 32]. Isotopic assay such as the tritium-labeled hypoxanthine assay allows rapid, sensitive, and accurate determination of parasite growth. This assay uses high degree of automatization and thus reduces variability that could be caused by human factors. Moreover, it also has been used for in vitro antimalarial drug sensitivity assay [31]. However, this assay has disadvantages, particularly due to the use of isotopic materials. The regulations on using and handling of radioactive compounds have become more restrictive since late 1970s due to the hazard that it may cause. Moreover, the technique is also considerably expensive with high cost of equipment such as scintillation counters and harvesting machine. Therefore, this isotopic assay has been replaced by more economical and safer alternative tests.

Fluorimetric assay with DNA-binding fluorescent dyes is a nonmorphological and nonradioactive assay [33]. This assay detects and measures the content of DNA of intact malaria-infected erythrocytes using specific fluorescent dyes that bind with the DNA [34]. This assay requires extra steps in its sample preparation to lyse the erythrocytes and to extract the DNA using chloroform in order to eliminate hemozoin that can cause quenching. This nonradioactive assay is simple, fast, and accurate with less expensive instrument use. However, the use of toxic dyes is a cause of concern. Ethidium bromide, for example, is a highly toxic and mutagenic compound that needs proper handling. Waste that contains this compound also requires specific and systematic disposal arrangement. Thus, this fluorometric assay should be revised and improvement needs to be made before this assay can be considered as an alternative standard method in this area of research.

An ELISA with monoclonal antibodies directed against either plasmodial lactate dehydrogenase (pLDH) or histidine-rich protein II (HRP II) which were commonly used in drug discovery strategies because of either of the two is practical and less hazardous compared to others.

*HRP II Testing*.* Histidine-rich protein* II (PfHRP2) is natural 30 kDa protein, part of cytoplasm of* P. falciparum, *heat stable which synthesized only by* P. falciparum* parasites [35]. This protein is very stable and had correlation between blood concentration of protein and parasite biomass [36]. According to [37] PfHRP2 was important factor in detoxification of heme. PfHRP2 was found in all strains of* P. falciparum* and in plasma and supernatant of culture as dissolved protein. PfHRP2 also was found on the surface of infected erythrocytes membrane. PfHRP2 contains 35% histidine, 40% alanine, and 12% aspartate but the percentages of these amino acids vary depends on the isolate [38].

*P. falciparum* that penetrates human erythrocyte will grow and divide into new parasite within 48 hours. This parasite ingests up to 80% of the host hemoglobin through a protozoan, phagocytic organelle known as the cytosome [39]. The hemoglobin is then transported into an acidic vacuole by the cytosome. In this vacuole, hemoglobin is broken down by proteolytic enzyme into small peptides to serve as nutrients for the parasite [40]. The degraded hemoglobin would form free toxic product as free heme. Later the free heme detoxication is changed by polymerization of free heme into inert hemozoin [41]

Screening using* P. falciparum* maintained in vitro could be determined by using the PfHRP2 assay. Through this assay, PfHRP2 production could be used as an indicator for the growth of the parasite and the increasing of parasite in total [42]. The screening of PfHRP2 cannot be used for prediction of parasite response to treatment since the parasite antigen still presents persistence in blood circulation after parasite clearance [43]. However, this method has been successfully used in assessing the antimalaria activity of dihydroartemisinin, mefloquine, quinine, and chloroquine [44].

*pLDH Assay*.* Plasmodium* Lactate Dehydrogenase (pLDH) is an energy-producing enzyme and the final enzyme in parasite glycolytic pathway. It is soluble and is produced by sexual and asexual stages of all four human* Plasmodium *species [45]. This assay detects pLDH antigen, which is the specific marker for the presence of* Plasmodium* in sample. Based on this principle, this assay could detect malarial pLDH enzyme which utilized 3-acetylpyridine nicotinamide adenine dinucleotide (APAD) as NAD and nicotinamide adenine dinucleotide analogue, whereas erythrocyte LDH could not [45, 46]. Pyruvate could be formed from L-lactate as product in the presence of pLDH enzyme and APAD as coenzyme. This reaction produces a reduced form of APAD which in turn reduces nitro blue tetrazolium, a blue formazan product. This product could be detectable both visually and by spectrophotometer at 650 nm [34]. In addition to enzymatic reaction described, two monoclonal antibodies specific for* P. falciparum *lactate dehydrogenase (pLDH) were used to develop a double-site enzyme-linked LDH immunodetection assay (DELI) or in vitro assay. As a result, the DELI was a useful and practical method to determine the differential parasite growth in the presence of increasing drug concentrations, for example, to develop a simple colorimetric drug sensitivity assay [47], whereas munumbicin D [23] and kakadumycin A [25] are the example of antimalarial compounds that have been successfully tested using this method. This assay is useful for monitoring antimalarial therapy as well as to differentiate* P. falciparum* and other* Plasmodium* spp. [46].

#### 4.1.2. *In Vivo* Study

*Peter's Test*. Four-day suppressive test which is also known as Peter's test is testing the efficacy of four daily doses of compounds. The testing is measured by comparing of blood parasitemia (on day four after infection) and mice survival time in treated and untreated mice [48]. The most widely used parasite is* Plasmodium berghei* [5]. This test is used to determine the antimalarial activity of candidates on early infections [49].

The infection of rodent could be initiated through needle passage from infected mice to naïve mice via intraperitoneal route using a small inoculum in range between 10^6^ and 10^7^ infected erythrocytes. Then, compound under study was administered through several routes such as intraperitoneal, intravenous, subcutaneous, or oral route. Recently, Gancidin-W, an antimalarial compound was successively tested using this methods [26].

*Rane's Test*. The purpose of this test is to evaluate the curative capability of candidate drug on established infections [49]. This test measures the ability of a standard inoculum of* P. berghei *to kill the recipient mice within 6 days of inoculation. Extension of survival beyond 12 days is determined to have a positive activity [50].

Each of the mice received a standard inoculum intravenously and treatment was withheld for 72 h to allow parasitaemia to establish. Blood smears were made from the tail blood of each mouse on five consecutive days, starting from the day of treatment. Average percentage parasitaemia was assessed. The number of deaths was also recorded for 28 days and the mean survival time is obtained afterward. Previous study has successfully been conducted by using this method in testing the antimalarial activity by combining two compounds, which were cryptolepine and artemisinins [51].

## 5. Metabolomics Platform

Metabolomics approach is critically dependent on technologies to identify and characterize chemical entities [52]. Three methods can be utilized for metabolomics platform which are Liquid Chromatography-Mass Spectrophotometry (LC-MS), Gas Chromatography-Mass Spectrophotometry (GC-MS), and Nuclear Magnetic Resonance (NMR) spectroscopy.

LC-MS platform is a very useful tool in analysing a wide range of semipolar compounds including many secondary metabolites of interest [53]. The coupling of liquid chromatography to mass spectrometers with high mass resolution (typically > 5,000 full width at half maximum (FWHM)) and high mass accuracy (typically < 5 ppm) provides high chromatographic resolution with high mass accuracy for detection of putative metabolite identification. Furthermore, LC-MS provides detection of higher-molecular-weight compounds of medium-to-high lipophilicity, including many classes of lipids (glycerolipids, phospholipids, fatty acids, bile acids, and sterols). Besides, sample preparation for LC-MS analysis only involved deproteinization, lyophilization, and reconstitution in a suitable aqueous/organic solvent mixture [54]. LC-MS provides a versatile tool to undergo the majority of analytical tasks in metabolite profiling studies.

Otherwise, GC-MS is used to analyse the volatile organic compounds (VOCs) [53]. GC-MS methods provide the detection of low-molecular-weight metabolites with a boiling point (either before or after chemical derivatisation) low enough to allow elution through a GC column. The boiling points of these metabolites are typically below 300°C. Metabolites detectable using this method include amino and organic acids, fatty acids, carbohydrates, phosphorylated metabolites (such as glucose-6-phosphate), and cholesterol [54]. GC-MS is easy to operate and is a low cost instrument. However, the sample preparation is tedious, time-consuming, and prone to many errors. Nonvolatile metabolites may be converted into different forms of derivatives during the derivatisation reaction, thus producing a specimen where different forms of the same parent metabolite exist together. In addition, there is an issue of by-product formation and degradation. Depending on the conditions, metabolites are derivatised with different conversion rates [55]. Moreover, inaccurate quantification also might occur due to incomplete derivatisation. However, there are some strategies which can be applied to tackle this such as by using both derivatised standard compounds and by applying data correction strategies to normalize the bias. GC-MS by its nature is limited to the analysis of small volatile molecules and molecules that can be made volatile [56].

Proton nuclear magnetic resonance (^1^H-NMR) spectroscopy is one of the most popular methods in metabolomics approach. This technique is useful for the analysis of bulk metabolites [53]. It requires simple and nontedious sample preparation steps and produces reproducible result. This method has low sensitivity compared to other methods and only allows for the detection of the most abundant metabolites. Besides that, the cost to run the sample by NMR instrument is also relatively high. As a result availability of NMR spectroscopy and time available for this instrument for this type of work are both limited [56].

## 6. Conclusion and Future Perspectives

The metabolomics is a great opportunity, as this technique provides extra knowledge and information for the analysis of natural products and it provides dereplication of drugs redundancy to shorten the long process in drug discovery. In addition, this technology helps to identify and to optimize the production of secondary metabolites. The coupling with the other “omics” can be made with the help of chemometrics or bioinformatics. In general, metabolomics approach in malaria drug discovery especially in Malaysia is still new and needs to be initiated. As this technique offers a new dimension of research, it could maximise the potential of discovering novel therapeutic antimalaria compounds from the natural product especially in drug research in Malaysia.

## Figures and Tables

**Table 1:** List of antimalarial agent sources and their biological compounds.

Actinobacteria	Compound	Testing approach	Reference
*Streptomyces ochraceus* and *Streptomyces bottropensis*	Trioxacarcins (Trioxacarcin A, B C D)	In vitro testing	[20]
*Streptomyces *sp. MSU-2110	Coronamycins	In vitro testing	[21]
*Streptomyces* NRRL 3052	Munumbicins, E-4 and E-5	In vitro testing	[22]
*Streptomyces* NRRL 30562	Munumbicin D	In vitro testing	[23]
*Streptomyces spectabilis *BCC 4785	Metacycloprodigiosin	In vitro testing	[24]
*Streptomyces* sp. NRRL 30566	Kakadumycin A	In vitro testing	[25]
*Streptomyces* sp. SUK 10	Gancidin-W	In vivo testing	[26]
*Streptomyces albidoflavus*	Antimycin A18	In vitro testing	[27]
*Streptomyces* sp. CS,	bafilomycin A_1_	In vitro testing	[28]
